# Adherence to iron with folic acid supplementation and its associated factors among pregnant women attending antenatal care follow up at Debre Tabor General Hospital, Ethiopia, 2017

**DOI:** 10.1371/journal.pone.0210086

**Published:** 2019-01-07

**Authors:** Alemayehu Digssie Gebremariam, Sofonyas Abebaw Tiruneh, Bedilu Abebe Abate, Melaku Tadege Engidaw, Desalegn Tesfa Asnakew

**Affiliations:** Department of Public Health, College of Health Sciences, Debre Tabor University, Debre Tabor, Ethiopia; B P Koirala Institute of Health Sciences, NEPAL

## Abstract

**Introduction:**

Nutritional anemia is a major public health problem throughout the world, particularly in developing countries. Iron with folic acid supplementation (IFAS) is recommended to mitigate anemia and its resulting complications during pregnancy. There has been limited study on IFAS adherence of pregnant women in the study area. The aim of this study was to assess adherence to IFAS and its associated factors among pregnant women attending antenatal care service in Debre Tabor General Hospital, Ethiopia.

**Methods:**

An institution-based cross-sectional study was conducted from January 9 to April 8, 2017, at Debre Tabor General Hospital. A total of 262 study participants were included and selected by systematic random sampling. The entire interviewed questionnaire was checked and entered into EpiData version 3.1 and then exported to SPSS version 20 for windows for analysis. IFAS adherence status was defined as, if pregnant mothers took 65% or more of the IFAS which is equivalent to taking IFAS at least 4 days a week during the 1-month period preceding the study. Regressions were fitted to identify independent predictors of IFAS adherence. A P-value of less than 0.05 was used to declare statistical significance.

**Results:**

A total of 241 pregnant women were included (92% response rate), of which 107 (44%) were adherent to IFAS. Only 39% received IFAS counseling, and 52% had some knowledge of IFAS. Gravidity (AOR = 2.92 95% CI (1.61, 5.30)), gestational age at first ANC visit (AOR = 3.67, 95% CI (1.94, 6.97)), pregnant women who got advice about IFAS (AOR = 2.04, 95%CI (1.12, 3.75)), current anemia (AOR = 2.22, 95%CI (1.45, 4.29)), and had knowledge about IFAS (AOR = 3.27, 95% CI (1.80, 5.95)) were statistically associated with adherence to IFAS among pregnant women.

**Conclusion:**

Overall, IFAS adherence among pregnant women was low. The associated factors with adherence of IFAS were counseling and knowledge, early ANC attendance, pregnancy history, and current anemia diagnosis. IFAS counseling by health workers was low but, when given, was associated with improved IFAS adherence. Health workers and health extension workers should consistently counsel on IFAS benefits during ANC visit, to improve IFAS adherence during the current and subsequent pregnancies.

## Introduction

Nutritional anemia is a major public health problem throughout the world, particularly in developing countries and evidence suggests that iron deficiency is the most common cause of nutritional anemia in the world [1,2]. Anemia during pregnancy diagnosed as hemoglobin concentration less than 11 g/dl for mild anemia, between 7–9.9 g/dl for moderate anemia and less than 7g/dl considered as severe anemia and usually caused by iron deficiency [3].

Globally, 1.62 billion people suffered from anemia that accounts for 24.8% of the world’s population. It is estimated that 56.4 million pregnant women affected by anemia and Africa account for 17.2 million pregnant women affected by anemia [4]. According to the WHO 2012 report, more than 40% of worldwide pregnant women were anemic. Among this, 50% of the burden is caused by iron deficiency [5]. In Ethiopia, 31.7% of pregnant women were anemic [6], and other several studies in Ethiopia reviled that there is a high prevalence of anemia during pregnancy [7–9]. According to 2016 Ethiopian demographic health survey report, 24% of reproductive age group women was anemic [10]. Overall, anemia contributes to 18% of perinatal mortality, 19% of preterm births, and 12% of low birth weight in low and middle-income countries [11].

Anemia during pregnancy has a significant adverse effect both on the mother and fetus [12]. So far, micronutrient deficiency of iron and folic acid during pregnancy are the most risk factors for anemia and this contributes to the poor neonatal health and increased maternal mortality [13]. Iron and folic acid is the most prevalent micronutrient deficiency in the world particularly among pregnant women especially in developing countries [14].

Daily 30mg to 60mg of iron and 0.4mg folic acid is highly recommended for pregnant women to prevent maternal anemia, puerperal sepsis, low birth weight, neural tube defect, and preterm birth. Daily iron and folic acid supplementation (IFAS) during pregnancy reduced the risk of all types of maternal anemia at term by 70% and iron deficiency anemia at term by 57% [5]. According to 19 African countries, national demographic health survey dataset analysis evidenced that, IFAS during pregnancy for 90 days can reduce the risk of neonatal mortality by 34% [15,16].

In Ethiopia, several studies evidenced that there is poor adherence of prenatal IFAS during antenatal period [17–19] but there is no study to show the magnitude of IFAS in worldwide and Africa. Ethiopian demographic health survey of 2016 showed that only 5% pregnant mothers took an iron with a folic acid tablet for 90 days and 58% of pregnant mothers did not take any iron with a folic acid tablet during their time of pregnancy [10]. Poor adherence of IFAS is associated with low utilization of antenatal care services, inadequate supply of IFA tablets, poor counseling and lack of knowledge on anemia [20,21]. However, IFAS is highly recommended for anemia control and prevention [22] but in Ethiopia, the adherence and coverage to IFAS are still very low. [10]. Furthermore, there are limited studies conducted to address the issue of adherence of IFAS and its associated factors in the study area. Therefore, the result of this study will provide invaluable information on IFAS adherence and its associated factors for policy makers and health planners.

The aim of this study was to assess adherence to IFAS and its associated factors among pregnant women in Debre Tabor General Hospital.

## Methods and participants

### Study setting and subjects

An institution-based cross-sectional study was conducted in Debre Tabor General Hospital from January 09- April 08/2017. Debre Tabor General Hospital is found in Debre Tabor Town, South Gondar Zone of Amhara Regional state which is about 667 kilometers away from Addis Ababa in Northwest direction and 102 kilometers far from Bahir Dar. The Hospital offers health services including maternal and child health services. Some of these are inpatient, outpatient, neonatal intensive care, TB/ leprosy, antenatal care, delivery, postnatal care, and family planning services.

All pregnant women who had at least two visits of Antenatal care for current pregnancy at Debre Tabor General Hospital and previously supplemented with 60mg iron with 0.4mg folic acid tablets for at least one month before the date of interview was included while pregnant women with mental disorder, unable to hear and/or speak, and very sick were excluded from the study.

A total of 262 study participants were included into this study after adding 5% of none response rate. This sample size was calculated by using single population proportion formula by considering the expected prevalence of adherence to IFAS among pregnant women was 20.4% from the study done in Mecha district [18], 95% of confidence level, and 5% of marginal error. Systematic random sampling was employed to select the study participants. First, we estimate the number of ANC service users for the previous three consecutive months which was 1502. Then, we calculated the K^th^ interval and which was 6. Finally, we interviewed the study participants at every six intervals among ANC service users.

### Data collection procedure

The data collectors were 5 diploma nurses. The data were collected by using face to face interview and chart review through structured interviewer-administered questionnaire under the supervision of 2 health officers. The questionnaire was developed after a review of the different kind of literature. The questionnaire was first developed in English. This questionnaire translated into Amharic and then translated back into English by a different person to check its consistency. Two days training was given for data collectors and supervisors. The questionnaire was pretested on 5% of the sample size before the actual data collection period. The training was given for the data collectors and supervisors. All pregnant women were checked for anemia as ANC client and we assessed the anemia by reviewing clients chart. The filled questionnaires were checked for completeness and consistency by the supervisors.

### Operational definitions

**Adherence to IFAS:** Mothers were said to adhere to IFAS if they took 65% or more of the supplement, equivalent to taking the supplement at least 4 days a week during the 1-month period preceding the study [23].

**Good Knowledge About IFAS:**—Those who score above 4 questions prepared to assess comprehensive knowledge of IFAS of the respondents [23].

### Data processingand analysis

All the interviewed questionnaires were checked manually for completeness and consistency. Data were coded and entered by using EpiData version 3.1 software. Double entry was made. The entered data were exported into the Statistical Package for Social Science (SPSS) version 20 software for further analysis. The analysis was conducted in several steps. First, simple descriptive statistics such as a frequency distribution and percentages were performed to describe the demographic, socioeconomic, and obstetric characteristics of the respondents. Second, a bivariate logistic regression was performed for each independent variable with the outcome variable. A variable whose bivariate test had a p-value <0.2 was a considered as a candidate for the multivariable model. Once the variables were identified, multivariable analysis was conducted. Hosmer and Lemeshow’s goodness-of-fit test was used to check the appropriateness of the data for multiple logistic regression. Finally, a multivariable logistic regression model was done to determine independent predictors of adherence status to IFAS. Crude and adjusted odds ratios together with their corresponding 95% of confidence intervals were computed to see the strength of association. All tests were two-sided and P < 0.05 was considered to declare statistical significance. Finally, the result was presented by tables.

The study was conducted after getting an ethical clearance letter from the institutional ethical review committee of Debre Tabor University. The data were collected after obtaining a permission letter from the Debre Tabor General Hospital. Informed consent was secured from each study participants and personal identifiers were excluded during the data collection to assure confidentiality.

## Results

### Socio-demographic characteristics of study participants

A total of 241 study participants were involved with a response rate of 92%. A large proportion of the study participants were within the age range of 25–29 years (35%) and almost all were orthodox (91%) religion followers. Majority of the respondents were married (69%) and having a family size of 4–6 (61%) as shown in the table below (Table 1).

**Table 1:** Socio-demographic characteristics of pregnant women attending ANC service at Debre Tabor General Hospital, North West Ethiopia, 2017.

Variables		Frequency	Percent
Age	15–19	10	4.1
	20–24	64	26.6
	25–29	85	35.3
	30–34	62	25.7
	≥35	20	8.3
Religion	Orthodox	220	91.3
	Muslim	12	5
	Protestant	9	3.7
Educational level	Unable to read and write	63	26.1
	Can read and write	51	21.2
	Primary education	36	14.9
	Secondary education	56	23.2
	College and above	35	14.5
Occupation	Housewife	87	36.1
	Daily laborer	31	12.9
	Government employ	77	32.0
	Merchant	46	19.1
Marital status	Married	167	69.3
	Single	31	12.9
	Divorced	33	13.7
	Widowed	10	4.1
Family size	1–3	33	13.7
	4–6	147	61.0
	≥ 6	61	25.3
Time elapsed from Home to Hospital	<30 minutes	144	59.8
	≥30 minutes	97	40.2
Total		241	100

### Reproductive health characteristics of study participants

Out of 241 study participants, 55% had an experience of more than one pregnancy. A large proportion of pregnant women (49%) were Nullipara. About seventy-four percent (74%) of respondents were in their third trimester of the pregnancy during the data collection period. Majority of study participants (65%) came before 16 weeks of gestational age for their first ANC visit. Seventy-three percent of pregnant women attended three to four ANC visit whereas the rest had only two ANC visits. Majority of the study participants (61%) had not been advised about the use of IFAS. Most pregnant women (81%) had a history of anemia in their lifetime and 28% of pregnant women had anemia currently. Nearly half (52%) of the study participants were knowledgeable about IFAS (Table 2).

**Table 2:** Obstetrics and health-related characteristics of pregnant women attending ANC service at Debre Tabor General Hospital, North West Ethiopia, 2017.

Variables		Frequency	Percent
Gravidity	Primigravida	109	45.2
	Multigravida	132	54.8
Parity	Nullipara	105	43.6
	Primipara	63	26.1
	Multipara	73	30.3
Living child	No	106	44.0
	Yes	135	56.0
Trimester	Second	63	26.1
	Third	178	73.9
ANC visit < 16 weeks	No	85	35.3
	Yes	156	64.7
Number of ANC visit	Two	65	27.0
	Three to four	176	73.0
Advised about IFAS	No	147	61.0
	Yes	94	39.0
History of Anemia	No	194	80.5
	Yes	47	19.5
Anemia currently	No	174	72.2
	Yes	67	27.8
Knowledge about IFAS	No	117	48.5
	Yes	124	51.5
IFAS adherence	No	134	55.6
	Yes	107	44.4

### Associated factors of adherence to IFAS

In bivariate logistic regression gravidity, parity, having child, gestational age at first ANC visit, getting advice about IFAS, current anemia, and knowledge about IFAS were significantly associated with adherence to IFAS during pregnancy.

In multivariable logistic regression gravidity, gestational age at first ANC visit, getting advice about IFAS, current anemia, and knowledge about IFAS were significantly associated with adherence to IFAS during pregnancy. Multigravida mothers were 2.92 times more likely to be adherent to IFAS than Primigravida mothers (AOR = 2.92, 95%CI (1.61, 5.30)). Pregnant women who had first ANC visit before 16 weeks of gestational age were 3.67 times more likely to be adherent to IFAS than those pregnant women who had first ANC visit after 16 weeks of gestational age (AOR = 3.67, 95% CI (1.94,6.97)). Mothers who got advice about IFAS were 2.04 times more likely to be adherent to IFAS than those who didn’t get advice (AOR = 2.04, 95%CI (1.12, 3.75)). Pregnant mothers who had anemia currently were 2.22 times more likely to be adherent to IFAS than those pregnant mothers who don’t have anemia (AOR = 2.22, 95%CI (1, 15, 4.29)). Pregnant women who had knowledge about IFAS were 3.27 times more likely to be adherent to IFAS than those who didn’t have knowledge (AOR = 3.27, 95% CI (1.80, 5.95)) as shown in the table below (Table 3).

**Table 3:** Associated factors of IFAS among pregnant womena ANC service at Debre Tabor General Hospital, North West Ethiopia, 2017.

Variables		Adherence status		COR (95% CI)	AOR (95%CI)
		Non-Adherent	Adherent		
Gravidity	Primigravida	78	31	1	1
	Multigravida	55	76	3.40(1.99, 5.86)	2.92(1.61, 5.30)
Parity	Nullipara	73	32	1	
	Primipara	29	34	2.68 (1.40, 5.15)	
	Multipara	32	41	2.92 (1.57, 5.44)	
Having children	No	73	33	1	
	Yes	61	74	2.68 (1.58, 4.57)	
ANC visit < 16 weeks	No	64	21	1	1
	Yes	70	86	3.74 (2.09, 6.72)	3.67 (1.94, 6.97)
Advised about IFAS	No	92	55	1	1
	Yes	42	52	2.07(1.22, 3.51))	2.04 (1.12, 3.75)
Current Anemia	No	108	66	1	1
	Yes	26	41	2.58 (1.45, 4.60)	2.22 (1.15, 4.29)
Knowledge about IFAS	No	81	31	1	1
	Yes	53	71	3.01 (1.77, 5.12)	3.27 (1.80,5.95)

## Discussion

Nutritional anemia is a major public health problem throughout the world, particularly in developing countries. Pregnant women are one of the vulnerable populations to develop iron deficiency anemia. The World Health Organization recommends IFAS to all pregnant women a standard dose of 30mg–60mg iron and 400μg folic acid daily throughout pregnancy [5]. Pregnant women adherence to IFAS plays a major role in the prevention and treatment of iron deficiency anemia. The aim of this study was to assess adherence to IFAS and its associated factors among pregnant women in Debre Tabor General Hospital.

The result of this study revealed that 44% of pregnant women were adherent to IFAS which is consistent with a study done at Egypt (41%), Misha district (39%) and Bahir Dar (42%) [24–26]. On the other side, it is higher than the study done at Uganda (12%), Kenya (33%), Goba, Southeast Ethiopia (18%) and Mecha district (20%) [17,18,27,28]. The possible reasons might be the study setting and time which means our study was Hospital-based and recent whereas their studies were community-based. It was lower than the study conducted at South India (64%), Akaki Kality, Addis Ababa (60%), Gondar, Northwest Ethiopia (55%), Assella (60%) and Mizan Aman (71%) [29–33]. The possible justification might be urbanization, accessibility to health services and awareness towards IFAS.

This study showed that gravidity had a significant association with adherence to IFAS. Multigravida mothers were 2.92 times more likely to be adherent to IFAS than Primigravida mothers. This is consistent with the study done in India and Assella [30,31] whereas it is not similar to the study done at Kenya and Misha district [25,27], which showed that gravidity had no association with IFAS adherence. The possible justification could be multigravida mothers might have higher knowledge, awareness, and experience of nutritional anemia due to their repeated visit to health facilities.

Our study indicated that gestational age at first ANC visits is a predictor of adherence to IFAS. Pregnant women who had first ANC visit before 16 weeks of gestational age were 3.67 times more likely to be adherent to IFAS than those pregnant women who had first ANC visit after 16 weeks of gestational age. It is in line with the study done at the Western Zone of Tigray, Assella and Mizan Aman [19,30,33]. The possible justification might be pregnant women who registered early for ANC service could acquire a better knowledge of perceived risk and benefit of IFAS to prevent anemia during pregnancy.

In addition, our study presented that advice about IFAS had statistical significance with IFAS adherence status. Pregnant women who got advice about IFAS were 2.04 times more likely to be adherent to IFAS than those who didn’t get advice. This is similar to the study done at Uganda, Misha district and Mizan Aman [25,28,33]. The possible reason may be getting advice may increase the level of knowledge, attitude and practice towards IFAS adherence.

The present study revealed that anemia during current pregnancy is a predictor of adherence to IFAS. Pregnant mothers who had anemia currently were 2.22 times more likely to be adherent to IFAS than those pregnant mothers who didn’t have anemia. It is consistent with the study conducted in the northwestern zone of Tigray, India, and Mecha district [18,19,31]. The possible justification may be the perceived risk of complication of anemia may be high in pregnant women who have current anemia.

Furthermore, this study indicated that knowledge about IFAS has an association with adherence to IFAS. Pregnant women who had knowledge about IFAS were 3.27 times more likely to be adherent to IFAS than those who didn’t have knowledge about IFAS. It is similar to the study dine at Mecha district, Misha district, and Goba district [17,18,25]. The reason may be knowledge will increase the level of awareness about IFAS and in turn, it will increase the attitude and practice towards adherence of IFAS.

### Limitation of the study

All knowledge item questions answers were yes and the data collectors were nurses. So, these may introduce social desirability bias. The other limitation of this study is we didn’t assess the side effects of the iron with folic acid tablet but it may have an impact on the adherence rate of IFAS.

### Conclusion and recommendation

Adherence of IFAS among pregnant women was low. Gravidity, gestational age at first ANC visit, advice about IFAS, current anemia, and knowledge about IFAS were independent predictors of adherence to IFAS. Health professionals shall provide health information for all ANC service users in the regular base during counseling session of ANC visit about the benefit of IFAS to prevent maternal anemia and its consequence by enhancing adherence to IFAS. Ministry of Health and its stakeholders shall mobilize the community to bring pregnant women to health facility early (before 16 weeks of gestational age) for ANC service and deliver health education to enhance the awareness of mothers towards IFAS to increase the adherence status of pregnant women to IFAS.

## Supporting information

S1 Table SPSS data for IFA adherence. (SAV)

S2 Table Questionnaire in English version. (DOCX)

S3 Table Questionnaire in Amharic version. (DOCX)
