# Supplementary material for: Adherence to iron with folic acid supplementation and its associated factors among pregnant women attending antenatal care follow up at Debre Tabor General Hospital, Ethiopia, 2017
Source: PLoS One. 2019 Jan 7;14(1):e0210086. doi: 10.1371/journal.pone.0210086 (PMC6322725; doi:10.1371/journal.pone.0210086)
Supplement: S2 Table — (DOCX) [file pone.0210086.s002.docx]

**Appendix**

**Participant information sheet and informed consent form for pregnant women (English version)**

**Hello! My name is** ________and I am collecting data for a study on the **Adherence to Iron with Folic Acid Supplementation and Its Associated Factors among Pregnant Women Attending Antenatal Care Follow up at Debre Tabor General Hospital, Ethiopia, 2017.** I would like to ask you some questions about the associated factors of adherence to iron with folic acid supplementation. The interview would take about 25 minutes. Your participation in the study is very critical to know the magnitude of Adherence to iron with folic acid and its associated factors. However, your participation is fully voluntary. The data you provided will be kept in a highly confidential manner and none of your **personal identifiers will be on the questionnaire**.

**Part one: Socio Demographic and Economic Characteristics**

| S. No | **Question** | **Choices of response** |
| --- | --- | --- |
| 101 | Age | ------------------ |
| 102 | What is your religion? | 1. orthodox  2.Muslim  3. protestant  4. other specify |
| 103 | What is your educational status? | 1 Cannot read and write  2. Write and read  3. Primary education  4. Secondary education  5.Other |
| 104 | What kind work do you work? | 1. housewife  2.daily laborer  3.government employee  4.merchant 5. other specify |
| 105. | What is your marital status? | 1. married  2. single  3. divorced  4. widowed |
| 106. | What your family size? | 1. 1- 3 2. 4-6  3. Above 6 |
| 107. | What is the average monthly income of your family? | ---------------? |

**Part two: health and health facility related questions**

| 108 | How many times you were pregnant | ------------------? |
| --- | --- | --- |
| 109 | Do you have a child (children)? | 1.Yes  2.No |
| 110 | How many children do you have | ------------? |
| 111 | What is gestational age now (chart review) | -------------? |
| 112 | When did you visit the ANC clinic during this pregnancy | 1.<4 months  2. > months |
| 113 | How many ANC visit do you have? | 1.One to Two  2.Three to Four  3.> four |
| 114 | how much time it takes from your home to the hospital (by foot in a minute) | -------------? |
| 115 | During any of your antenatal care visit did you get advice about IFAS from health professionals. | 1. Yes  2. No |

**Part Three: knowledge about iron and folic acid supplementation**

| 116. | Have you ever heard about IFAS? | 1.Yes  2.No |
| --- | --- | --- |
| 117 | Taking IFAS during pregnancy is it important to the mother? | 1. Yes  2. No |
| 118 | Taking IFAS during pregnancy is it important to the fetus? | 1. Yes  2. No |
| 119 | Do you think taking IFSA starts from confirmation of pregnancy and continue throughout pregnancy? | 1. Yes  2. No |
| 120 | Do you think that taking IFSA during pregnancy is it important to prevent anemia | 1. Yes  2 No |
| 127 | Do you think iron and folic acid tablet continue at postpartum period? | 1. Yes 2. No |
| 128 | Taking iron and folic acid tablets during pregnancy don’t lead to too big baby | 1. Yes 2. No |
| 129 | Taking iron and folic acid tablets during pregnancy may help to prevent birth defects | 1. Yes 2. No |

**Part four: IFAS Adherence and other health related characteristics**

| 122 | Do you have a history of anemia? | 1.Yes  2.No |
| --- | --- | --- |
| 123 | Do pregnant mother has anemia during current pregnancy? (chart review) | 1.Yes  2.No |
| 126 | How many tablets did you take on average per week | -----------? |

**THANK YOU VERY MUCH!!!**
