# Supplementary material for: Adherence to iron with folic acid supplementation and its associated factors among pregnant women attending antenatal care follow up at Debre Tabor General Hospital, Ethiopia, 2017
Source: PLoS One. 2019 Jan 7;14(1):e0210086. doi: 10.1371/journal.pone.0210086 (PMC6322725; doi:10.1371/journal.pone.0210086)
Supplement: S3 Table — (DOCX) [file pone.0210086.s003.docx]

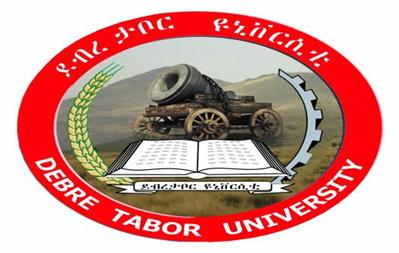


ደብረ ታቦር ዩኒቨርሲቲ

የጤና ሳይንስ ኮሌጅ

የህብረተሰብ ጤና ትምህርት ክፍል

የተጠያቂው / መላሾች የመረጃ ቅፅ

እንደምን አደሩ / ዋሉ ፡፡ ስሜ ------------------------------- እባላለው:: ከዚህ የመጣሁት በደብረ ታቦር ዩንበቨርስቲ የህብረተሰብ ጤና ት/ት ክፍል መምህራን የሆኑትን የእነ አለማየሁን ወከዬ ነው፡፡እርስዎ የተመረጡት በዚህ ተቋም የወሊድ ክትትል በማድረግ ላይ ስለሚገኙ ብቻ ነው፡፡ የእርስዎ ተሳትፎ ሙሉ በሙሉ በእርስዎ ፈቃደኝነት ላይ የተመሰረተና በጥናቱ መሳተፍ ያለመሳተፍ መብት አለወዎት፡፡ መጠይቁ በአጠቃላይ እስከ 25 ደቂቃ ሊወስድ ይችላል፡፡

ክፍል አንድ: አጠቃላይ መረጃ

| **ተ.ቁ** | **መጠይቅ** | **ምላሽ/ አማራጭ** |
| --- | --- | --- |
| 101 | እድሜ | ------------------ በድፍን አመት |
| 102 | ሀይማኖት | 1. ኦርቶዶክስ 2. ፕሮቴስታንት 3. ሙስሊም 4. ካቶሊክ 5. ሌላ ካለ ይጠቀስ _______________ |
| 103 | የትምህርት ሁኔታ | 1. ማንበብ እና መፃፍ የማይችል  2. ማንበብ እና መፃፍ የሚችል  3. የመጀመሪያ ደረጃ ትምህርት የተማረ  4. ሁለተኛ ደረጃ ትምህርት የተማረ  5. ሌላ ካለ ይጠቀስ |
| 104 | የስራ ሁኔታ | 1. የቤት እመቤት 2. የቀን ሰራተኛ 3. የመንግስት ሰራተኛ 4. ነጋዴ 5. ሌላ ካለ ይጠቀስ_____________ |
| 105. | የጋብቻ ሁኔታ | 1. ያገባ 2. ያላገባ 3. የተፋታ 4. ባሏ የሞተበት |
| 106. | የቤተሰብ መጠን | 1. 1- 3 2. 4-6 3. ከ 6 ቤተሰብ በላይ |
| 107. | አማካኝ የወር ገቢ | _____________________? |

**ክፍል ሁለት፡ ጤና እና የጤና ሁኔታን የተመለከቱ መጠይቆች፡፡**

| **ተ.ቁ** | **መጠይቅ** | **ምላሽ/ አማራጭ** |
| --- | --- | --- |
| 108 | ለምን ያክል ጊዜ አርግዘሽ ታውቂያለሽ፡፡ | ------------------? |
| 109 | ልጅ አለዎት፡፡ | 1.አዎ  2.የለኝም |
| 110 | ስንት ልጅ አለዎት፡፡ | ------------? |
| 111 | አሁን እርግዝናዎ ስንተኛ ዕድሜው ነው (ቻርት በማየት) | -------------? |
| 112 | ለእርግዝና ክትትል ወደ ጤና ድርጅት የሄዱት መቸ ነበር፡፡ | 1. ከአራት ወር በፊት  2. ከአራት ወር በኋላ |
| 113 | በእርግዝናዎ ወቅት ለስንት ጊዜ የእርግዝና ክትትል ነበተረዎት፡፡ | 1. ከአንድ እስከ ሁለት ጊዜ 2. ከሶስት እስከ አራት ጊዜ 3. ከአራት ጊዜ በላይ |
| 114 | በእግር መንገድ ከቤትዎ ጤና ድርጅት ድረስ ስንት ሰዓት ይወስዳል፡፡ | -------------? |
| 115 | ለእርግዝና ክትትል ሲመጡ በእርግዝና ጊዜ ስለሚወሰድ የአይረን(የደም ማነስ መከላከያ ትምህርት) ተምረዉ ያውቃሉ፡፡ | 1. አዎ 2. አልወሰድኩም |

**ክፍል ሶስት፡ ስለ አይረን ( የደም ማነስ መከላከያ) ግንዛቤን የሚመለከቱ መጠይቆች፡፡**

| **ተ.ቁ** | **መጠይቅ** | **ምላሽ/ አማራጭ** |
| --- | --- | --- |
| 116. | ስለ አይረን (የደም ማነስ መከላከያ) ሰምተው የሚያውቁት ነገር አለ? | 1. ስምቸ አውቃው 2. ሰምቸ አላውቅም |
| 117 | አይረን (የደም ማነስ መከላከያ) በእርግዘና ጊዜ መውሰድ ጠቃሚ ነው ብለው ያስባሉ? | 1. አዎ 2. አይጠቅምም |
| 118 | አይረን (የደም ማነስ መከላከያ) በእርግዘና ጊዜ መውሰድ ለፅንስ ጠቃሚ ነው ብለው ያስባሉ? | 1. ይጠቅማል 2. አይጠቅምም |
| 119 | አይረን (የደም ማነስ መከላከያ) የሚወሰደው እርግዝና መኖሩ ከተረጋገጠ በኋላ ሙሉ በእርግዝና ወቅት የሚወሰድ ነው ብለው ያስባሉ? | 1. አዎ 2. አላስብም |
| 120 | አይረን (የደም ማነስ መከላከያ) በእርግዝና ጊዜ መውሰድ የደም ማነስን ይከላከላል ብለው ያስባሉ? | 1. አዎ 2. አላስብም |
| 121 | አይረን (የደም ማነስ መከላከያ) ከወሊድ በኋላ ይወሰዳል ብለው ያስባሉ? | 1. አዎ 2. አላስብም |
| 122 | አይረን (የደም ማነስ መከላከያ) በእርግዝና ጊዜ መውሰድ የፅንሱ ክብደት ይጨምረዋል ብለው ያስባሉ፡፡ | 1. አዎ 2. አላስብም |
| 123 | አይረን (የደም ማነስ መከላከያ) በእርግዝና ጊዜ መውሰድ በፅንሱ ላይ የሚደረስ የእርግዝናን የአካል ጉዳት ይቀንሳል ብለው ያስባሉ፡፡ | 1. አዎ 2. አላስብም |

**ክፍል አራት፡ አይረን (የደም ማነስ መከላከያ) አወሳሰድን የሚመለከቱ መጠይቆች**

| **ተ.ቁ** | **መጠይቅ** | **ምላሽ/ አማራጭ** |
| --- | --- | --- |
| 124 | በህይወት ዘመንዎ የደም ማነስ ህመም ነበረበዎት፡፡ | 1. አዎ 2. የለብኝም |
| 125 | ነፍሰ ጡር እናቷ የደም ማነስ ችግር አለባት፡፡(ቻርት በማየት) | 1. አዎ 2. የለባትም |
| 126 | በአማካኝ በሳምንት ስነት የአይረን (የደም ማነስ መከላከያ) ክኒን ይወስዳሉ፡፡ | -----------? |

እናመሰግናለን!!!
